# V‐PRO Blood Collection Tubes: Validation for Clinical Chemistry and Immunoassay Tests

**DOI:** 10.1002/jcla.70007

**Published:** 2025-03-04

**Authors:** Anwar Borai, Wedyan Alsharif, Amirah Alhindi, Maha Alqahtani, Mohieldin Elsayid, Haitham Khalil, Salwa Al Marwani, Abobaker Yagoot, Janet Magjacot, Maha Al Meteiri, Rawan Alyamani, Hind Abdulhakim, Majid Al‐Thaqafy

**Affiliations:** ^1^ King Abdullah International Medical Research Center (KAIMRC) King Saud bin Abdulaziz University for Health Sciences (KSAU‐HS), King Abdulaziz Medical City, Ministry of National Guard Jeddah Saudi Arabia; ^2^ King Fahad Armed Forces Hospital Department of Pathology and Laboratory Medicine Jeddah Saudi Arabia

**Keywords:** blood, tube, vacutainer, validation, V‐PRO

## Abstract

**Background:**

In accredited laboratories, each component of diagnostic products—such as laboratory instruments, reagents, and blood collection tubes must be validated before integration into routine patient testing. BD Vacutainers are commonly used in clinical laboratories compared to other blood collection tubes, while V‐PRO tubes have recently been introduced to the market without prior laboratory validation. This study compares V‐PRO tubes to BD Vacutainers to assess the validity of using V‐PRO tubes for blood testing.

**Materials and Methods:**

Blood samples were collected simultaneously into two different brands of tubes (V‐PRO and BD) from 60 subjects. A standardized procedure was employed for sample collection, and analysis. A total of 28 chemistry tests and 20 immunoassays were analyzed using Abbott instruments, while high‐performance liquid chromatography was used for testing glycated hemoglobin. The biases of V‐PRO compared to BD were evaluated against current desirable quality specifications for bias derived from biological variation. For technical validation, a designated survey was distributed to various institutes using both tube types in their laboratories.

**Results:**

The V‐PRO tube exhibited biases exceeding the desirable limits for CO_2_ (3.2%), magnesium (2.0%), thyroid‐stimulating hormone (11.7%), and estradiol (−8.5%). Survey results indicated a higher percentage of major pre‐analytical, analytical, and post‐analytical errors when using the V‐PRO tube compared to the BD Vacutainer.

**Conclusions:**

Laboratories currently using BD vacutainers should exercise caution if they intend to perform chemistry and immunoassay tests with V‐PRO tubes. The technical validation outcomes for V‐PRO were not acceptable due to significant faults identified in comparison to BD Vacutainer.

## Graphical abstract

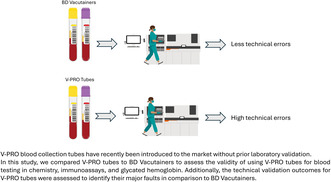

## 1 Introduction

The preanalytical phase is a crucial step in diagnostic clinical laboratory. Consequently, various errors—such as improper sample collection, mislabeling, inadequate sample transportation, and poor‐quality blood tubes—can lead to inaccuracies in laboratory results that affect patients' lives [1, 2, 3, 4, 5, 6].

Accordingly, all collection tubes used in the laboratory must be validated for their effectiveness and reliability, especially since some new brands of blood collection tubes lack prior validation. Therefore, laboratories must conduct their own validation.

BD Vacutainers are currently used in our laboratory, while V‐PRO tubes have recently been introduced to the Saudi Arabian market and lack a history of prior validation.

Before V‐PRO tubes are officially integrated into routine blood collection, our preliminary observations indicate that they have several technical limitations during the preanalytical and analytical phases of our clinical chemistry automated system. Additionally, the impact of V‐PRO tubes on patient data is still unknown, as is the extent to which their inefficiencies may compromise the reliability of laboratory test results [7].

The aim of this study was to validate the V‐PRO tubes for serum separator (SST) and EDTA tubes. Additionally, a technical assessment survey was conducted to evaluate the performance of V‐PRO tubes compared to BD Vacutainers as used by different laboratory operators.

## 2 Methods and Materials

### 2.1 Subjects' Recruitment

Participants for the study were recruited from both healthy and non‐healthy individuals at King Abdulaziz Medical City, National Guard Hospital in Jeddah. The study received approval from the Institutional Review Board (IRB) at King Abdullah International Medical Research Center (KAIMRC) for ethical considerations (SP22J/105/08). Each participant signed an informed consent form after being informed about the study's purpose and assured that it posed no additional risks. The subjects consisted of 30% males, with ages ranging from 18 to 83 years and a mean of body mass index (BMI) ± standard deviation (SD) of 28.0 ± 5.3 kg/m^2^.

### 2.2 Blood Samples and Analysis

Blood sample collection and tube validation were conducted in accordance with Clinical Laboratory Standards Institute (CLSI) guidelines [8, 9, 10]. Blood samples were collected from 60 subjects after obtaining demographic details, including height, weight, age, and gender. Each subject provided a total of six blood tubes: three from BD Vacutainers (Becton Dickinson and Company, Franklin Lakes, NJ, USA)—two SST 5.0 mL (lot 2333400) and one EDTA 4.0 mL (lot 2143926)—and three from V‐PRO (Advance Medical Co., Riyadh, KSA)—two SST 5.0 mL (lot E1021) and one EDTA 4.0 mL (lot O0722). To prevent carryover contamination, SST tubes were collected first, followed by the EDTA tubes. Blood sample collection was conducted according to a randomized schedule.

Blood samples were drawn in the phlebotomy area of the outpatient clinic from 20 apparently healthy subjects and 40 subjects from different wards, including dialysis (10 subjects), emergency (10 subjects), oncology (10 subjects), and cardiac (10 subjects). A total volume of 20–25 mL of blood was collected from each volunteer. Venipuncture was performed daily from 8:00 to 10:00 AM over a two‐week period. All healthy volunteers fasted for approximately 12 h and were seated for 15 min prior to blood collection. Samples exhibiting lipemia, hemolysis, icterus, or intravenous contamination were excluded based on automated analysis indices criteria.

Preanalytical variables were standardized throughout the study, including blood collection procedures, mixing, transportation to the laboratory, and centrifugation. The same phlebotomists and laboratory specialists conducted all procedures throughout the study. EDTA samples were used for testing glycated hemoglobin (HbA1c), while serum separator tube (SST) samples were centrifuged (1200 g) for 10 min and specimen then analyzed within 1 h of collection using Abbott analyzers (Architect c8000 for chemistry tests and the Architect i2000 for immunoassays). HbA1c was analyzed immediately using the D‐100 System from Bio‐Rad based on high‐pressure liquid chromatography (HPLC) with ion exchange.

In total, 360 blood samples were collected from 60 volunteers: 180 samples using BD Vacutainers and 180 using new V‐PRO tubes. Samples were transported to the laboratory without delay and handled immediately upon receipt to minimize variability in results. SST tubes were utilized for 28 clinical chemistry tests and 20 immunoassays, while EDTA tubes were specifically used for HbA1c testing.

The clinical chemistry tests, immunoassays, glycated hemoglobin, their abbreviations, and principles of measurement are summarized in Table 1. To facilitate a technical comparison between the two tube types, a survey was designed to assess the technical aspects of both tubes by different phlebotomists and laboratory specialists. The survey questions focused on the pre‐analytical, analytical, and post‐analytical phases of testing. It was distributed to six laboratories across different health institutions that had prior experience or current exposure to both BD Vacutainers and V‐PRO tubes.

**TABLE 1:** List of tests with their principles of measurement.

Abbr	Chemistry Analyte (Architect c8000)	Method	Abbr	Immunoassay Analyte (Architect i2000)	Method
TP	Total protein	Biuret method	Ferritin	Ferritin	CMIA
Alb	Albumin	Bromcresol green	AFP	α‐fetoprotein	CMIA
Urea	Urea	Urease	CEA	Carcinoembryonic antigen	CMIA
UA	Uric acid	Uricase	CA125	Carbohydrate antigen 125	CMIA
CRE	Creatinine	Kinetic Alkaline Picrate	CA199	Carbohydrate antigen 199	CMIA
TBil	Total Bilirubin	Diazonium salt	CA153	Carbohydrate antigen 153	CMIA
Glu	Glucose	Hexokinase/G‐6‐PDH	PSA	Prostate specific antigen	CMIA
DBil	Direct Bilirubin	Diazo Reaction	TSH	Thyroid stimulating hormone	CMIA
TC	Total cholesterol	Cholesterol oxidase (Enzymatic)	FSH	Follicle‐stimulating hormone	CMIA
TG	Triglycerides	Glycerol Phosphate Oxidase	FT4	Free thyroxin 4	CMIA
HDL‐C	High density lipoprotein‐Cholesterol	Accelerator selective detergent	FT3	Free thyroxin 3	CMIA
LDL‐C	Low density lipoprotein‐Cholesterol	The Friedewald equation	LH	Luteinizing hormone	CMIA
Na	Sodium	Ion‐selective electrode diluted (Indirect)	PRL	Prolactin	CMIA
K	Potassium	Ion‐selective electrode diluted (Indirect)	Cort	Cortisol	CMIA
Cl	Chloride	Ion‐selective electrode diluted (Indirect)	Estradiol	Estradiol	CMIA
Ca	Calcium	Arsenazo‐III dye	PTH	Intact parathyroid hormone	CMIA
IP	Inorganic phosphorous	Phosphomolybdate	Insulin	Insulin	CMIA
Mg	Magnesium	Enzymatic (NADP to NADPH)	C‐pep	C‐peptide	CMIA
AST	Aspartate aminotransferase	NADH (without P‐5'‐P)	B12	Vitamin B12	CMIA
ALT	Alanine aminotransferase	NADH (without P‐5'‐P)	Folate	Folate	CMIA
LDH	Lactate dehydrogenase	Lactate to pyruvate	**Abbr**	Analyte (Biorad D‐100)	**Method**
ALP	Alkaline phosphatase	p‐nitrophenyl phosphate hydrolysis	HbA1c	Glycated hemoglobin	HPLC
GGT	Gamma glutamyl transferase	L‐Gamma‐glutamyl‐3‐carboxy‐4‐nitroanilide Substrate			
CK	Creatine Kinase	NAC (N‐acetyl‐L‐cysteine)			
Amy	Amylase	CNPG3 substrate			
CRP*4	C‐reactive protein	Immunoturbiditmetry			
Fe	Iron	Ferene‐S			
CO2	Bicarbonate	PEP Carboxylase			

Abbreviations: CMIA, Chemiluminescent microparticle immunoassay; HPLC, high pressure liquid chromatography.

Sample collection and analysis were conducted at King Abdulaziz Medical City (National Guard Hospital, Jeddah, Saudi Arabia), which is accredited by the College of American Pathologists and participates in a rigorous internal and external quality control program. In addition to healthy subjects, patients with various clinical conditions were included in the study to ensure that a range of concentrations for each test could be covered during the validation process.

### 2.3 Statistical Analysis

Statistical analysis was performed using SPSS software version 25. Non‐parametric statistical methods were applied in our study. Therefore, Spearman's correlation and Wilcoxon's analysis were employed to calculate the correlation coefficient (r) and assess differences between means. The statistical significance was defined as *p* < 0.05. The difference percentage between BD (X) and V‐PRO (Y) results for each test was calculated as “Y‐X/X*100”. Finally, the bias between V‐PRO and BD Vacutainer was compared with the current desirable quality specifications for bias (B), and derived from biological variation according to the formula B < 0.25 (CVw2 + CVg2)1/2 where CVw and CVg are within‐ and between‐subject CVs obtained from Ricos et al. [11]. The coefficient of variation (CV), which indicates precision, was calculated for each test in each tube by repeating the analysis twenty times within a single run. The CV was estimated using the following formula: ‘SD/mean * 100.’

## 3 Results

### 3.1 Patients' Comparison

The calculated means and standard errors (SE) for all chemistry and immunoassay tests, including HbA1c, are presented in Tables 2 and 3, respectively.

**TABLE 2:** V‐PRO (SST) tubes compared to BD (SST) vacutainers for chemistry tests.

Chemistry assay	Unit	V‐PRO			BD			* *r*	(BD) CV	(V‐PRO) CV	%bias	(B) Ricos et al. [11]
		Mean ± SE	Min	Max	Mean ± SE	Min	Max					
Amy	U/L	72 ± 5	24	244	72 ± 5*	24	244	0.999**	1.1	0.7	0.6	7.8
Ca	mmol/L	2.31 ± 0.01	1.99	2.59	2.29 ± 0.01***	1.96	2.52	0.873**	2	1.5	0.7	0.8
TC	mmol/L	4.5 ± 0.2	1.2	7.3	4.4 ± 0.2***	1.1	7.4	0.987**	0.6	2.4	2.0	4.1
DBil	umol/L	4.5 ± 0.6	1.9	32.7	4.6 ± 0.6**	1.9	33.7	0.963**	4.9	3.2	−2.7	14.2
Na	mmol/L	137.5 ± 0.4	127	145	137.9 ± 0.4	127	145	0.843**	0.4	0.6	−0.3	0.3
Glu	mmol/L	6.8 ± 0.4	4.1	20.1	6.7 ± 0.4**	4.1	18.8	0.965**	0.5	0.9	1.6	2.5
HDL‐C	mmol/L	1.3 ± 0.1	0.3	2.3	1.2 ± 0.1*	0.3	2.3	0.989**	0.9	0.9	1.2	5.2
ALK	U/L	86.6 ± 8.5	45	528	87.0 ± 8.5	45	531	0.990**	1	0.7	−0.3	6.4
ALT	U/L	17.4 ± 1.6	6.0	71.0	17.2 ± 1.6	6.0	74.0	0.983**	3.5	1.5	10.3	11.5
CO_2_	mmol/L	22.9 ± 0.3	15.0	31.0	22.3 ± 0.3*	17.0	31.0	0.719**	2.8	3.4	**3.2**	1.8
K	mmol/L	4.1 ± 0.1	3.2	5.0	4.1 ± 0.1	3.1	5.0	0.890**	1.1	0.6	0.9	1.8
Urea	mmol/L	8.3 ± 0.9	2.3	35.7	8.2 ± 0.9**	2.2	35.0	0.992**	3.5	1.4	1.8	5.5
AST	U/L	18.0 ± 0.8	8.0	40.0	17.8 ± 0.8	7.0	38.0	0.946**	2	2.5	1.4	5.4
Cl	mmol/L	102.4 ± 0.5	91.0	107.0	102.6 ± 0.6	90.0	108.0	0.844**	0.6	0.5	−0.3	0.5
CRP	mg/L	13.3 ± 3.4	0.3	150	13.0 ± 3.3***	0.2	147.5	0.999**	1.1	1.1	0.2	24.9
PO4	mmol/L	1.24 ± 0.03	0.83	2.34	1.26 ± 0.03***	0.85	2.34	0.978**	0.8	0.9	−1.6	3.2
TBil	umol/L	9.6 ± 1.0	3.5	50.1	9.4 ± 1.0*	3.5	50.3	0.990**	2.4	1.7	2.1	10
CK	IU/L	138 ± 37	20	2228	137 ± 37	18	2225	0.997**	2	4.1	1.0	11.5
CRE	umol/L	203 ± 9	44	1093	201 ± 39	45	1094.2	0.967**	1.9	2.2	2.0	3.4
UA	umol/L	320 ± 15	79	707	319 ± 15	79	707	0.997**	1.7	0.7	0.1	4.8
Alb	g/L	43 ± 1	30	51	43 ± 1.0***	29	50	0.967**	1.1	2.0	1.1	1.3
FE	umol/L	9.8 ± 0.6	1.6	20.1	9.7 ± 0.6	1.6	20.1	0.992**	1.3	0.6	0.1	8.8
LDH	U/L	185 ± 5	115	347	191 ± 5*	121	352	0.815**	1.1	3.1	−2.0	4.3
GGT	IU/L	35 ± 9	6	524	35 ± 9	6	524	0.991**	1.3	2.9	0.0	10.8
Mg	mmol/L	0.85 ± 0.02	0.56	1.37	0.83 ± 0.02***	0.50	1.34	0.935**	2.2	1.1	**2.0**	1.8
TP	g/L	73 ± 1	63	85	72 ± 1***	62	86	0.966**	0.7	0.5	0.9	1.2
TG	mmol/L	1.2 ± 0.1	0.45	5.26	1.2 ± 0.1***	0.38	5.16	0.992**	0.8	2.9	2.4	10.7
LDL‐C	mmol/L	2.7 ± 0.1	0.66	4.74	2.6 ± 0.1***	0.57	4.32	0.990**	3.9	3.9	2.7	6.8

Abbreviations: %bias, mean percentage difference; B, current desirable quality specifications for bias; *r*, correlation coefficient.

* *p* < 0.05.

** *p* < 0.01.

*** *p* < 0.001.

**TABLE 3:** V‐PRO tubes (SST, EDTA) compared to BD vacutainers (SST, EDTA) for immunoassay and HbA1c tests.

Immuno‐assay	Unit	V‐PRO			BD			* *r*	(BD) CV	(V‐PRO) CV	%bias	(B) Ricos et al. [11]
		Mean ± SE	Min.	Max.	Mean ± SE	Min.	Max.					
CEA	ng/mL	2.2 ± 0.2	0.5	4.7	2.2 ± 0.2*	0.64	5.9	0.968**	6.6	5.1	−3.4	14.1
Estradiol	pmol/L	136.8 ± 14.6	37.0	478.0	125.7 ± 14.6***	37.0	452.0	0.958**	6.7	12.6	**11.7**	8.3
PSA	ng/mL	0.05 ± 0.01	0.05	0.09	0.05 ± 0.01	0.05	0.09	0.999**	2.9	3.4	0.0	18.4
Ferrit	ug/L	23.6 ± 2.8	2	73	23.9 ± 2.87	2.0	73.0	0.988**	3.1	3.8	0.1	5.0
FT4	pmol/L	13.2 ± 0.2	9.0	17.4	13.1 ± 0.2	9.1	18.0	0.831**	2.2	3.2	0.5	3.6
CA199	IU/mL	8.6 ± 0.8	2.0	20.2	8.7 ± 0.8	2.1	19.8	0.958**	9.8	9.8	0.2	24
FT3	pmol/L	4.1 ± 0.1	2.7	5.3	4.2 ± 0.1	3.0	5.6	0.832**	4.7	2.8	0.0	4.8
PTH	pg/mL	81.6 ± 5.1	15.1	152.5	81.0 ± 5.1	16.3	160.1	0.988**	3.3	3.2	0.8	8.8
LH	IU/L	7.0 ± 0.8	0.1	19.0	7.0 ± 0.8	0.1	21.5	0.996**	2.8	5.7	−0.5	8.9
PRL	ug/L	13.7 ± 0.8	3.9	28.4	13.8 ± 0.8	3.9	28.6	0.996**	1.4	2.5	0.0	15.4
TSH	mIU/L	1.6 ± 0.2	0.2	5.8	1.8 ± 1.2***	0.2	5.1	0.992**	3.5	2.9	**−8.5**	8.4
CA125	IU/mL	14.5 ± 0.9	5.3	28.2	14.5 ± 0.8	5.7	27.8	0.993**	6.9	4.9	0.2	12.1
CA153	IU/mL	12.8 ± 0.6	6.1	22.1	12.9 ± 0.6	5.5	22.2	0.993**	5.1	2.8	0.2	10.8
Cort	nmol//L	241 ± 14	24	485	239 ± 14	20	509	0.987**	3.7	3.1	1.2	12.5
Folate	nmol/L	25.2 ± 0.9	11.7	41.3	25.2 ± 0.9	10.6	41.4	0.958**	3.8	4.7	1.0	19.9
AFP	ng/mL	2.4 ± 0.1	2.0	5.5	2.4 ± 1.0	2.0	5.5	0.983**	4.7	4.7	0.1	11.8
B12	pmol/L	314 ± 17	129	727	314 ± 16	105	667	0.960**	3.2	3.3	0.0	17.7
FSH	IU/L	7.3 ± 1.0	0.5	29.7	7.5 ± 1.0	0.5	29.1	0.993**	2.4	2.3	−1.0	8.4
Insulin	uIU/mL	15.8 ± 1.46	2.6	37.7	15.4 ± 1.49	0.2	38.9	0.951**	2.8	2.5	0.1	15.5
C‐pep	ng/mL	1615 ± 187	150	5724	1627 ± 188	144	5693	0.996**	3.2	3.0	−0.6	4.1
HbA1c	%	6.0 ± 0.2	3.8	10.9	6.0 ± 0.2	3.8	11.0	0.959**	2.1	1.6	−0.2	1.5

Abbreviations: %bias, mean percentage difference; B, current desirable quality specifications for bias; *r*, correlation coefficient.

* *p* < 0.05.

** *p* < 0.01.

*** *p* < 0.001.

The correlation coefficient (r) between the same tests conducted in both V‐PRO and BD tubes was significant for all chemistry and immunoassay tests (*p* < 0.01). For chemistry tests, the minimum r values were 0.719 for CO_2_, 0.815 for LDH, 0.843 for Na, 0.844 for Cl, 0.873 for Ca, and 0.890 for K. The maximum r values reached 0.999 for CRP and amylase; 0.992 for TG, urea, and iron; 0.991 for GGT; and 0.990 for LDL‐C, TBIL, and ALK (see Table 2).

For immunoassay tests, the minimum r values were 0.831 for FT4 and 0.832 for FT3, while the maximum r values were 0.999 for PSA; 0.996 for C‐pep, PRL, and LH; 0.993 for FSH, CA 15–3, and CA 125; and 0.992 for TSH (see Table 3).

The mean results for chemistry tests using V‐PRO were significantly different from those obtained with BD Vacutainers: *p* < 0.001 for Ca, TC, CRP, PO4, Alb, Mg, TP, TG, and LDL‐C; *p* < 0.01 for DBil, Glu, and Urea; and *p* < 0.05 for Amy, CO2, TBil, and LDH. For immunoassays, significant differences were observed only for CEA (*p* < 0.05) and Estradiol & TSH (*p* < 0.001).

### 3.2 Bias

The calculated percentage bias (% bias) for all tests is presented in Tables 2 and 3. Most of the obtained % biases were below the current desirable bias (B) derived from Ricos criteria for biological variation [11]. This indicates that the results obtained using V‐PRO tubes were acceptable for most tests when compared to those from BD tubes. However, this was not the case for two chemistry tests: the % bias for CO_2_ (3.2%) and Mg (2.0%) exceeded their respective desirable B limits of 1.8%.

Similarly, two immunoassays showed significant deviations, with % biases for Estradiol (11.7%) and TSH (−8.5%) exceeding their desirable B limits of 8.3% and 8.4%, respectively. These findings suggest that the results for CO_2_, Mg, Estradiol, and TSH immunoassays may be significantly affected by using the V‐PRO (SST) tube compared to BD (SST) Vacutainer.

**FIGURE 1:**
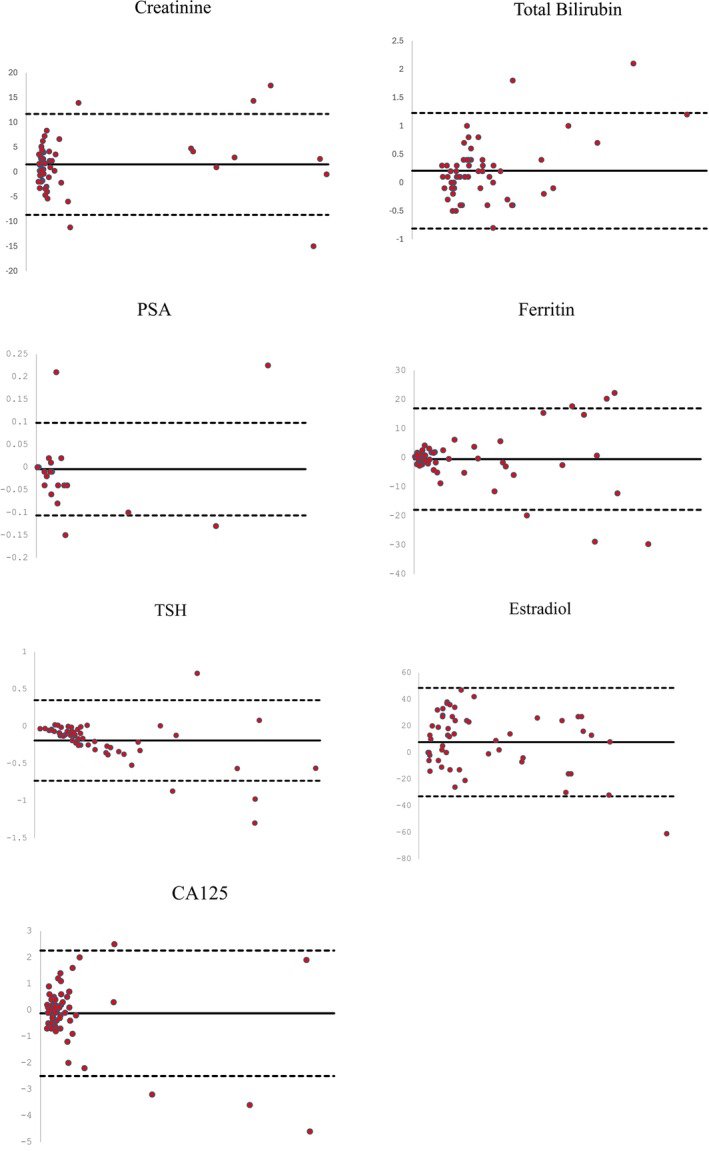
Bland–Altman plot for test results obtained from 60 subjects using V‐PRO tubes and BD Vacutainers for chemistry assays (creatinine and total bilirubin) as well as immunoassays (PSA, ferritin, TSH, estradiol, and CA125). The solid line represents the mean, while the dashed lines indicate the lower and upper limits (bias ±1.96*SD). The difference between the results from both tubes (V‐PRO and BD) is shown on the *y*‐axis, with the mean of both tubes readings on the *x*‐axis.

The Bland–Altman plot displays the differences between the results from the two tubes for each test on the y‐axis and the mean of the two measurements on the x‐axis [13]. The Bland–Altman plot analysis showed that the results obtained using the V‐PRO tube agree with those from the BD Vacutainer for all chemistry tests; however, variations at high levels can be observed for the chemistry tests of TBIL and CRE. Similarly, for immunoassays, variations at high levels can be observed for PSA, Ferritin, TSH, Estradiol, and CA125 (Figure 1).

### 3.3 Precision

Precision analysis was performed by using freshly collected blood samples from two healthy male and female subjects. Precision analysis was conducted for each test using both tubes by repeating the tests twenty times within a single run. The precision outcomes, expressed as % coefficients of variation (CV), for the V‐PRO tubes were comparable to those of BD Vacutainers, with both demonstrating values lower than those claimed by the commercial manufacturer in the kit insert for each test (Tables 2 and 3). The highest %CV observed was for the Estradiol test, which was 12.6%.

### 3.4 Survey

The survey consisted of 18 questions, with 9 focused on BD Vacutainers and the same 9 questions on V‐PRO tubes. These questions addressed common issues encountered during the preanalytical, analytical and postanalytical phases associated with each tube type. The survey was distributed to six central laboratories across various health institutes in Saudi Arabia, all of which have extensive experience using both brands of tubes, including serum separator, EDTA, and sodium citrate tubes.

A total of 15 responses were received from different staff members with varying laboratory proficiencies. The aim was to gather insights from personnel with diverse experiences, covering the preanalytical, analytical, and postanalytical phases. Four staff members were categorized as Group 1 (G1), performing both phlebotomy and laboratory specialist tasks in their laboratories. Five staff members were categorized as Group 2 (G2), working exclusively as phlebotomists, while Group 3 (G3) included six staff members who worked solely as laboratory specialists. Group 1 can address questions related to the preanalytical, analytical, and postanalytical phases; Group 2 can answer only preanalytical questions; and Group 3 can respond to analytical and postanalytical questions only. A summary of these responses is presented in Table 4.

**TABLE 4:** Survey summary of technical responses from six different institutes (15 total responses).

		BD vacutainer			V‐PRO tube		
Questions	Answers	G1	G2	G3	G1	G2	G3
Pre‐analytical phase							
1. If the tube has a clotting problem immediately after blood sample collection, how can you rate the occurrence of this incidence?	Very rare	100%	60%		25%	20%	
	Rare					20%	
	Sometimes		40%		25%	60%	
	Common				50%		
	Very common						
2. In case the tube has low blood flow while collecting the blood sample (low blood flow), how can you rate the occurrence of this incidence?	Very rare	75%	80%		25%	40%	
	Rare	25%					
	Sometimes		20%		50%	40%	
	Common				25%	20%	
	Very common						
3. If the pneumatic tube transportation system (PTS) is used in your laboratory, have you noticed any defect in the tube after blood sample transportation?	Yes		20%		25%	20%	17%
	No	50%	80%	50%	25%	60%	33%
	We don't have PTS	50%		50%	50%	20%	50%
4. How do you rate the incidence of broken blood sample in the centrifuge?	Very rare	100%		67%	75%		67%
	Rare			33%			33%
	Sometimes						
	Common				25%		
	Very common						
Analytical phase							
5. While the blood sample in the automation system is moving, how do you rate the incidence of error in the automated sample decapper?^a^	Very rare	75%		100%	25%		50%
	Rare	25%					17%
	Sometimes				25%		17%
	Common				25%		17%
	Very common				25%		
6. During the analysis of the tube by the automation system, how do you rate the incidence of error during sample aspiration?^b^	Very rare	75%		50%	25%		33%
	Rare	25%		50%	25%		33%
	Sometimes						17%
	Common				50%		17%
	Very common						
7. Using the tube, what kind of the most common technical limitations do you have?	Preanalytical	25%		17%	50%		34%
	Analytical	25%		17%	50%		67%
	Postanalytical						
	None of the above	50%		67%			17%
8. How can you rate the technical limitation of this tube on the laboratory workflow?	None	50%		67%	25%		17%
	Small	50%					
	Moderate			33%	50%		67%
	Major				25%		17%
Post‐analytical phase							
9. Do you have any idea if the tube can interfere with any of the laboratory test results? If yes, please specify which tests.	Yes				25% (Ca, Mg)		33% (PT, PTT, K)
	No	50%		33%			
	I don't know	50%		67%	75%		67%

Abbreviations: K, potassium; PT, prothrombin‐time; PTT, Partial thromboplastin time.

^a^ The automated sample decapper cannot completely remove the sample cap (rubber stopper) of the SST tube. The decapper removes the outer plastic piece but not the rubber stopper inside the tube.

^b^ Due to this error with the sample decapper, the probe may be damaged by the rubber stopper during the sample aspiration process.

In the pre‐analytical phase, 50% of Group 1 (G1) reported clotting problems after immediate sample collection using V‐PRO tubes as “common”, while 60% of Group 2 (G2) indicated similar issues with the same tube with the answer of “sometimes”. Additionally, 25% of G1 and 20% of G2 cited low blood flow as “common” when using V‐PRO tubes. G1 also reported that 25% of responses indicated incidents of broken samples in the centrifuge as “common” when using V‐PRO tubes.

In the analytical phase, G1 reported a 25% incidence of error in the automated sample decapper as “very common”, while 50% noted errors during sample aspiration as “common”. The most common technical limitations were identified as preanalytical by 67% of G3 respondents for V‐PRO tubes, compared to 17% for BD Vacutainers from the same group. Regarding technical limitations on laboratory workflow, 25% of G1 and, 17% of G3 reported as “major” by using V‐PRO tubes.

In the postanalytical phase, 25% of G1 indicated that V‐PRO tube usage interfered with laboratory results for calcium and magnesium, and 33% noted interference for potassium (K), prothrombin time (PT), and partial prothrombin time (PTT) with the same brand of tube.

## 4 Discussion

The validation process is essential in accredited clinical laboratories [14]. Total quality in diagnostic laboratories is important for in vitro diagnostic (IVD) companies. Therefore, they usually provide dedicated support and collaboration with customers to ensure that the total quality of their products is successfully achieved [15].

Components of blood collection tubes, such as surfactants, stoppers, stopper lubricants, separator gels, and clot activators, can interact with blood and consequently affect laboratory testing results [12]. As a result, many studies have been published on the validation of new brands of blood collection tubes or their components [16, 17, 18, 19].

BD vacutainers are a well‐known brand commonly used in clinical laboratories. In this study, we used BD vacutainers as a control to validate a new brand of blood collection tubes released on the market, named V‐PRO. To date, and to the best of our knowledge, there have been no validation studies on V‐PRO tubes; therefore, the outcomes of our validation are important for laboratory managers and quality officers when deciding whether to use this new product.

In this study, we validated 28 chemistry tests, 20 immunoassays, and HbA1c. We observed that the bias limit for desirable quality specifications was exceeded for the tests of CO_2_, magnesium, estradiol, and TSH. Therefore, switching to the V‐PRO tube may have a significant clinical impact on medical decisions regarding these tests based on our findings. For example, when measuring serum CO_2_ levels, it is important to note that switching from BD Vacutainer SST tubes to V‐PRO tubes could lead to clinically significant differences. This change may result in diagnostic errors, particularly for patients with renal impairment, electrolyte imbalances, acidosis, or alkalosis [20]. Additionally, CO_2_ is a sensitive test that can be easily affected by evaporation and sample stability; therefore, this may contribute to the differences in readings between the two types of tubes.

Magnesium (Mg) plays a critical role in energy metabolism, enzyme functions, and the regulation of parathyroid hormone (PTH) synthesis, release, and action [21]. Low Mg levels have been linked to impaired myocardial contractility and hypotension and are considered markers of atherosclerotic and vascular diseases [22]. Additionally, magnesium sulfate is important in treating eclampsia [23]. Inappropriately high Mg levels due to changes in vacuum tube brands (from BD to V‐PRO) could lead to misdiagnosis of patients.

Estradiol is used to assess female fertility, including the function of the ovaries, placenta, and adrenal glands. Additionally, monitoring estradiol levels during fertility therapy is important for evaluating follicular growth and treatment effectiveness [24]. The use of V‐PRO tubes may falsely elevate estradiol levels compared to BD vacutainers, which could have significant implications for patient diagnosis and treatment.

TSH (thyroid‐stimulating hormone) is produced by the anterior pituitary and serves as the primary stimulus for the production of thyroid hormones by the thyroid gland. Along with T3 and T4, it is essential for assessing whether thyroid disease is primary or secondary. Measuring TSH in conjunction with T3 and T4 is critical for diagnosing thyroid disorders and differentiating between their primary and secondary causes [25]. Therefore, laboratories using V‐PRO tubes may observe lower TSH levels compared to those obtained with BD vacutainers, potentially leading to patient misdiagnosis or ineffective treatment management.

In addition, our results indicate that the observed biases for Ca, Na, and Alb (0.7%, −0.3%, 1.1%) were at the threshold of the desirable quality specifications for bias [11]. Therefore, such tests should be carefully monitored during use or validation. However, all other test results in this study fell within the acceptable bias limits for desirable quality specifications and showed no clinically significant differences between V‐PRO and BD Vacutainer.

The reason for exceeding the bias limit of quality specification (B) for CO_2_, Mg, and TSH is unclear, whether it is due to the vacuum components, such as the plastic materials, gel matrix, or other factors. Additionally, the within‐run precision (CV) for Estradiol using the V‐PRO tube (12.6%) is higher than that of the BD Vacutainer, indicating that some interference affects estradiol measurement with the V‐PRO tube, leading to both an increased bias limit (B) and reduced precision.

The Bland–Altman plot analysis showed an acceptable systematic bias across the range of results for all chemistry and immunoassay tests. In some cases, the Bland–Altman plot indicated that the results obtained using the V‐PRO tube agreed better with those from the BD Vacutainer at low concentrations, but not at high concentrations. Variations at high concentrations were observed for TBIL, CRE, PSA, ferritin, TSH, estradiol, and CA125. These differences between the two tubes emphasize the importance of careful interpretation of results for these tests when using V‐PRO tubes at high concentrations compared to BD Vacutainers. In such cases, repeating the test is recommended.

The technical survey outcomes indicated that the V‐PRO tubes have significant limitations in the pre‐analytical, analytical, and post‐analytical phases compared to different types of blood collection tubes using BD Vacutainers. In the pre‐analytical phase, V‐PRO tubes exhibited a higher incidence of spontaneous blood clotting during sample collection. Additionally, the occurrence of weak negative pressure, which slows blood flow during collection and is typically noted by phlebotomists, was more common with V‐PRO tubes than with BD Vacutainers.

In the analytical phase, the survey revealed that errors in sample decapping were more frequent with the V‐PRO SST tube than with the BD Vacutainer, where such errors were very rare. From our experience, this represents a major technical fault, as it can damage the sampling probe of main analyzers and disrupt laboratory automation, significantly affecting workflow. This malfunction was reported as moderate by 50% and major by 25% of G1 users of the V‐PRO (SST) tube, while G3 reported it as “moderate” by 67% and “major” by 17%. These technical faults may be attributed to the dimensions of the V‐PRO tubes in comparison to BD Vacutainers using the automated system.

In conclusion, this study demonstrates that V‐PRO tubes are not an acceptable alternative to BD Vacutainers for certain chemistry and immunoassay tests, due to their significant technical limitations.

This study has several limitations. While we investigated the most common routine laboratory tests, other tests such as coagulation, serological, therapeutic, and molecular tests were not included. Another limitation is that our technical survey focused solely on SST, EDTA, and Sodium Citrate (blue top) tubes; other tubes, such as lithium heparin (green top) and fluoride oxalate (grey top), were not included in our survey or analysis and warrant further investigation. Additionally, our study was conducted using Abbott Architect and Bio‐Rad D‐100 analyzers. Therefore, our findings should be verified with other laboratory diagnostic analyzers and their automation system.

## Author Contributions

Anwar Borai, Wedyan Alsharif, and Amirah Alhindi were involved in the investigation and data collection. Maha Alqahtani, Mohieldin Elsayid, Majid Al‐Thaqafi and Salwa Al Marwani were project organizers. Anwar Borai was the principal investigator and designed the project and wrote the manuscript. Abobaker Yagoot, Janet Magjacot were co‐principal investigators. Haitham Khalil, Maha Al Meteiri, Hind Abdulhakim and Rawan Alyamani were the technical laboratory officers.

## Ethics Statement

Was obtained from King Abdullah International Medical Research Center with IRB number SP22J/105/08.

## Consent

We hereby confirm that each participant in this study signed an informed consent before participation.

## Conflicts of Interest

The authors declare no conflicts of interest.

## Data Availability

All data in this study on which the conclusions of the manuscript rely are available upon request from the corresponding authors for researchers who meet the criteria.
